# Microarray and Degradome Sequencing Reveal MicroRNA Differential Expression Profiles and Their Targets in *Pinellia* *pedatisecta*

**DOI:** 10.1371/journal.pone.0075978

**Published:** 2013-09-25

**Authors:** Miao Dong, Dongfeng Yang, Qiulei Lang, Wei Zhou, Shaowei Xu, Tao Xu

**Affiliations:** 1 College of Life Sciences, Zhejiang Sci-Tech University, Hangzhou, China; 2 LC Sciences, Hangzhou, China; Institut Pasteur of Shanghai, Chinese Academy of Sciences, China

## Abstract

MicroRNAs (miRNAs) are endogenous small non-coding RNAs which play a critical role in gene regulation in plants. *Pinellia* *pedatisecta* is one of the most important herbs in traditional Chinese medicine, but there are no microRNAs of *Pinellia* *pedatisecta* were deposited in miRBase and the research of the related miRNA biological functions is still insufficient. To detect *Pinellia* *pedatisecta* miRNAs and discover their expression difference with *Pinellia* *ternata* , we carried out a microarray profiling. A total of 101 miRNAs belonging to 22 miRNA families were detected both in *Pinellia* *pedatisecta* and *Pinellia* *ternata* respectively, among them 21 miRNAs showed their differentially expression. GO (gene ontology) term enrichment analysis of the target genes of differential expression miRNAs reveal that these miRNAs mainly affect the reproduction, transcription factor activity and plant developmental process. To elucidate the target function of miRNAs, we constructed a degradome library from *Pinellia* pedatisecta leaf. The result showed that a total of 18 transcript were identified as targets of miRNAs and further analysis indicated that miR156 and miR529 may function together to repress SPL14.

## Introduction

MicroRNAs (miRNAs) are endogenous ~21-nucleotide (nt) non-coding RNAs derived from single-stranded stem-loop RNA precursors; these RNA molecules regulate gene expression by guiding targeted mRNA cleavage or translational inhibition at the transcriptional and post-transcriptional levels [1,2]. Mature miRNAs are incorporated into the RNA-induced silencing complex (RISC), which regulates the expression of the complementary target mRNAs via the Argonaute (AGO) endoribonucleases [3]. Increasing evidence demonstrates that miRNAs are important for biological processes including developmental regulation, hormone response, leaf polarity establishment, morphogenesis, and stress adaptation [4,5,6]. Given that plant miRNAs usually have near-perfect complementary to their target sites in the protein-coding regions of mRNAs, most plant miRNAs function like siRNAs that guide target mRNA cleavage [7]. To date, miRNA microarray analysis is a rapid method for detecting and profiling a large number of miRNAs. Aside from its use in *Arabidopsis thaliana* [8] and *Oryza sativa* [9], miRNA microarrays have been successfully applied in other plant species, such as *Brassica napus* [10], *Solanum* *lycopersicum* [11], and *Medicago truncatula* [12]. Based on known miRNA sequences of a plant species, It may be used for plants with limited sequence information.

Plant miRNAs generally regulate gene expression by guiding targeted mRNA cleavage [13]. Thus, the key to completely understanding the biological functions of miRNAs is to discover their target genes. Computational prediction [14], AGO coimmunoprecipitation [15], and luciferase reporters [16] were always applied to identify the targets of miRNAs. However, these methods have several limitations, such as false positives during target gene prediction and the laborious methods for confirming predicted targets with their high cost and relatively small scale. Degradome sequencing is a novel approach based on next-generation sequencing; It can identify the target transcripts of miRNAs with high throughput [17]. The method involves deep sequencing, bioinformatics analysis, and 5'-rapid amplification of cDNA ends (5′-RACE) which has been successfully applied in the global identification of miRNA–target RNA pairs in *A. thaliana* [18], *O. sativa* [19], *Physcomitrella* *patens* [20], *Glycine max* [21], and *Zea mays* [22], among others.

*Pinellia* *pedatisecta* is one of the most important herbs in traditional Chinese medicine. This plant is a monocotyledonous perennial herbaceous species of the Araceae family native to Asia [23]. *P* *. pedatisecta* is characterized by a spathe without a constriction between the tube and blade. Compared with *P* *. ternate* which the properties and morphology are similar to other *Pinellia* species, *P* *. pedatisecta* contains pedate leaf blades and lacks a transverse septum inside its spathe [24]. In addition, *P* *. pedatisecta* tubers are commonly used as an antitussive, expectorant, and a anticancer drug, with relatively higher effectiveness than the tubers of other *Pinellia* species [25]. However, limited studies have focused on *P* *. pedatisecta* at the molecular level, especially its miRNA [24,26]. There are no microRNAs of *Pinellia* pedatisecta were deposited in miRBase and the research of the biological functions which the related miRNAs performed in *P* *. pedatisecta* is currently insufficient. This is the first report revealed miRNAs differential expression profile between *P* *. pedatisecta* and *P* *. ternate* and used degradome sequencing to identify the gene targets of *P* *. pedatisecta* miRNAs. In-depth analysis of the potential gene targets of miRNAs was performed to understand miRNA function in *P* *. pedatisecta* *.*

## Results

### Overview of *Pinellia* miRNA microarray

**Figure 1:**
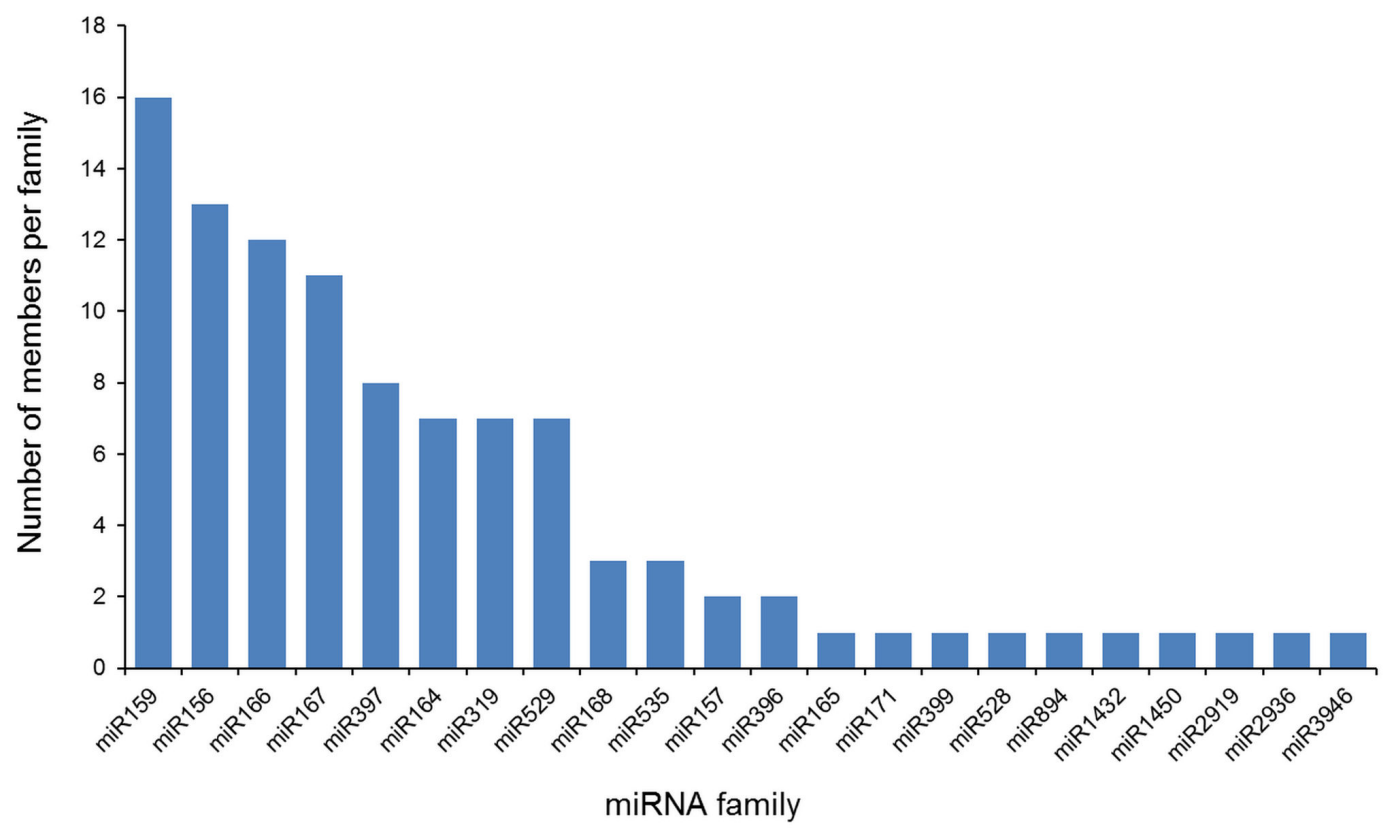
Number of miRNA members in each family which commonly identified from *P* *. pedatisecta* and *P* *. ternate* by microarray.

**Figure 2:**
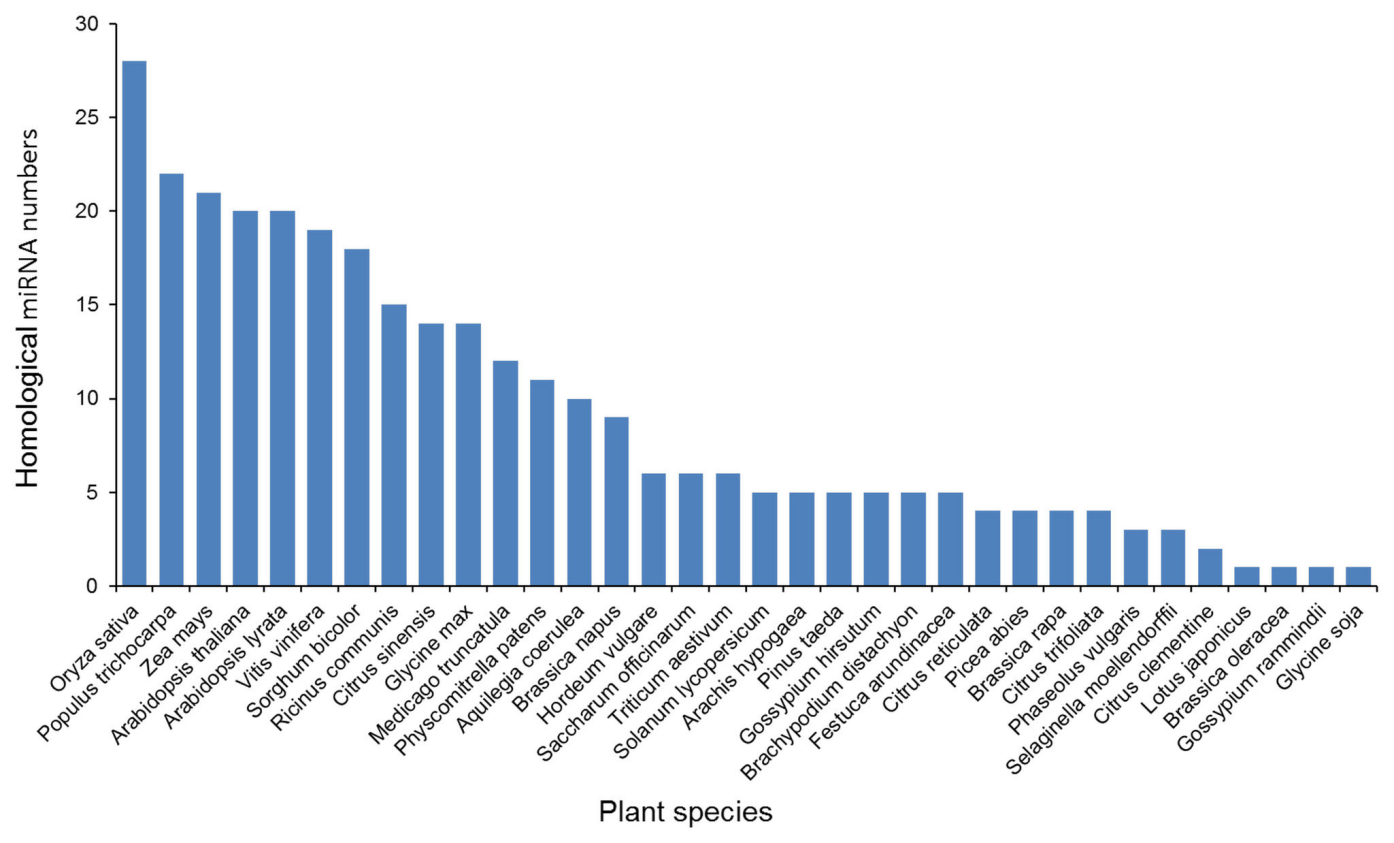
The homology of identified miRNAs in other plant species. Values on Y axis indicate the number of homological miRNA between *Pinellia* and other plant species.

To identify miRNAs in the *Pinellia* species, a miRNA microarray was used; this microarray contained probes complementary to known mature miRNAs from 43 plant species in the miRBase database [27]. The miRNA chip was hybridized with Cy5 and Cy3 probe pairs of *P* *. pedatisecta* and *P* *. ternata* , respectively. On the macro level, 101 unique plant miRNAs from 22 miRNA families were commonly identified from *P* *. pedatisecta* and *P* *. ternata* . Most of the identified miRNA families (miR159, miR156, miR166, miR167, and miR319) were highly conserved with numerous members, whereas several known but non-conserved miRNA families (miR528, miR894, miR1432, miR1450, miR2919, miR2936, and miR3946) had just one member (Figure 1) [28]. In addition, we investigated the homology between the identified miRNAs in this study and known miRNAs in other species. To a large extent, the *Pinellia* miRNAs were identified based on their high homology with *O. sativa* miRNAswhich considered a reference species for studying monocotyledons(Figure 2).

### Differential Expression of P. *pedatisecta* miRNAs

Although several dynamic factors influence miRNA function, the miRNA expression patterns were considered the major components that determine miRNA activity [29]. Thus, we compared the accumulation of known miRNA families using microarray signals to identify further the differential miRNA expression between *P* *. pedatisecta* and *P* *. ternata* . Based on a cut-off value and *p* < 0.01, 21 miRNAs from 12 families (Table 1) were differentially expressed. Among these, 14 miRNAs (from the miR156, miR166, miR168, miR397, miR528, miR535, and miR1450 families) were more abundant in *P* *. pedatisecta* (log_2_ fold change greater than 1), whereas the remaining 7 miRNAs (from the miR894, miR165, miR159, miR396, and miR2919 families) showed lower expression (log_2_ fold change less than −1). Previous studies reported that miR156, miR159, miR165/166, miR168, miR396, and miR397 participate in developmental regulation, transcription factor activity and stress adaptation by targeting SBP [30], MYB [31], the homeodomain-leucine zipper [32], AGO [33], growth response factor (GRF) [34], and laccase [35].

**Table 1:** Comparison of the expression patterns of miRNAs in *P* *. pedatisecta* and *P* *. ternata* leaves.

**miRNA family**	**miRNA Name**	**Sequence (5' to 3'**)	** *P* *. ternata* Signal-A**	** *P* *. pedatisecta* Signal-B**	**Log_2_ Ratio (B/A**)	**Predicted function of miRNAs**
miR156	ghr-miR156c	UGUCAGAAGAGAGUGAGCAC	101	1,192	3.56	Squamosa promoter-binding protein
	osa-miR156l	CGACAGAAGAGAGUGAGCAUA	202	1,594	2.98	
	vvi-miR156e	UGACAGAGGAGAGUGAGCAC	89	632	2.83	
	sbi-miR156e	UGACAGAAGAGAGCGAGCAC	298	2,105	2.82	
	bna-miR156a	UGACAGAAGAGAGUGAGCACA	468	3,200	2.77	
	aly-miR156g	CGACAGAAGAGAGUGAGCAC	538	3,295	2.61	
	aly-miR156a	UGACAGAAGAGAGUGAGCAC	557	3,401	2.61	
miR159	ptc-miR159f	AUUGGAGUGAAGGGAGCUCGA	7,570	3,150	-1.27	MYB transcription factors
	pta-miR159b	UUGGAUUGAAGAGAGCUCCC	3,465	1,265	-1.45	
	zma-miR159e	AUUGGUUUGAAGGGAGCUCCA	3,250	1,166	-1.48	
miR165	aly-miR165a	UCGGACCAGGCUUCAUCCCC	714	327	-1.13	
miR166	aly-miR166a-5p	GGAAUGUUGUCUGGCUCGAGG	64	722	3.49	
miR168	aly-miR168a-3p	CCCGCCUUGCAUCAACUGAAU	94	974	3.38	Polyadenylate-binding protein
	hvu-miR168-5p	UCGCUUGGUGCAGAUCGGGAC	427	1,027	1.27	
miR396	gma-miR396e	UUCCACAGCUUUCUUGAACUGU	2,078	731	-1.51	Growth regulating factor
miR397	ptc-miR397b	CCAUUGAGUGCAGCGUUGAUG	87	2,301	4.72	Laccase
miR528	osa-miR528	UGGAAGGGGCAUGCAGAGGAG	1,099	7,565	2.78	
miR535	aqc-miR535	UGACAACGAGAGAGAGCACGCG	9,629	19,234	1.00	
miR894	ppt-miR894	CGUUUCACGUCGGGUUCACC	4,403	2,105	-1.06	
miR1450	ptc-miR1450	UUCAAUGGCUCGGUCAGGUUAC	421	8,285	4.30	
miR2919	osa-miR2919	AAGGGGGGGGGGGGAAAGA	1,924	369	-2.38	

### miRNA validation by qRT-PCR

**Figure 3:**
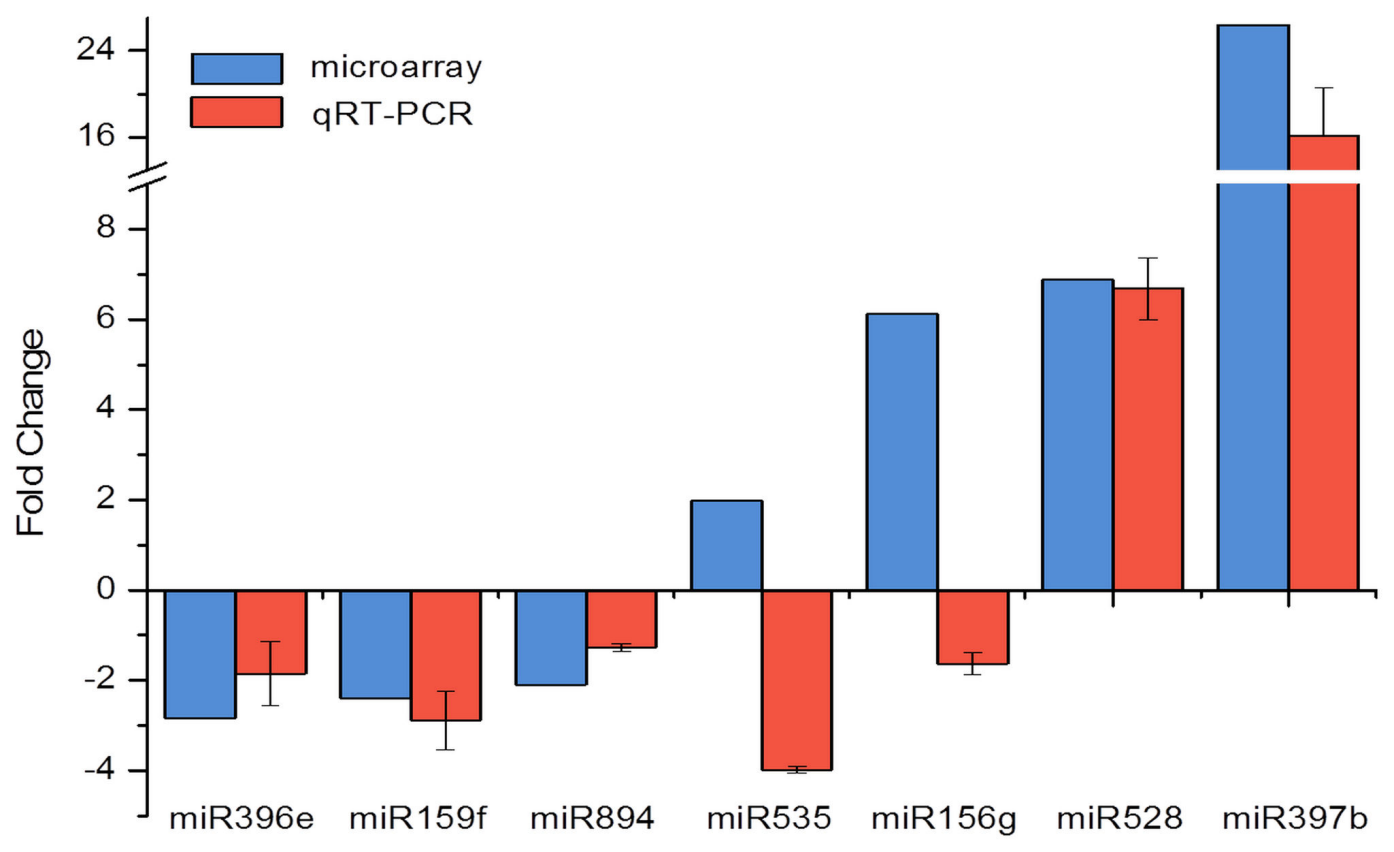
Validation and expression analysis of miRNAs by qRT-PCR (*P*.*pedatisecta*/*P*. *ternate*). Blue indicates the miRNA relative expression generated from microarray; Red indicates the miRNA relative expression tested by qRT-PCR. The 5.8s rRNA was chosen as the endogenous control. The error bars indicate the standard deviations obtained from three independent experiments of qRT-PCR.

qPCR was widely considered to be more accurate and quantitative methods for miRNA expression profiling than microarrays [36]. Based on the miRNA microarray results, 7 miRNAs differentially expressed in *P* *. ternata* and *P* *. pedatisecta* were selected for qRT-PCR analysis to confirm the presence and expression of the miRNAs. As shown in Figure 3, all miRNAs were detected by qRT-PCR and most results of qRT-PCR were consistent with the microarray data. In particular, miR159f, miR396e, and miR894 were more abundant in P. ternata, whereas miR528 and miR397b were more abundant in *P* *. pedatisecta* . However, the expression patterns of miR156g and miR535 differed between the microarray and qRT-PCR analyses. This finding may be attributed to the different sensitivity and specificity of the two technologies.

### Prediction of differentially expressed miRNA targets

Plant miRNAs usually regulate gene expression by guiding targeted mRNA cleavage [37]. Thus, the biological functions of miRNAs may be completely understood by discovering their target genes. To date, the *Pinellia* genome has yet to be sequenced, and no expressed sequence tags are available. According that conserved miRNA may regulated conserved targets [28], We used the Rice MSU RNA database (version 7.0) to computationally predict potential differentially expressed miRNA targets. Three computational target prediction algorithms (WMD3 http://wmd3.weigelworld.org/cgi-bin/webapp.cgi?page=TargetSearch;project=stdwmd, Targetfinder and psRNAtarget http://plantgrn.noble.org/psRNATarget/) were used. The Results revealed that 159 potential genes were targeted by 18 differential expression miRNAs. As shown in Table S1, 11 out of 18 miRNAs targeted transcription factors, thereby suggesting that these miRNAs mediate negative post-transcriptional regulation.

**Figure 4:**
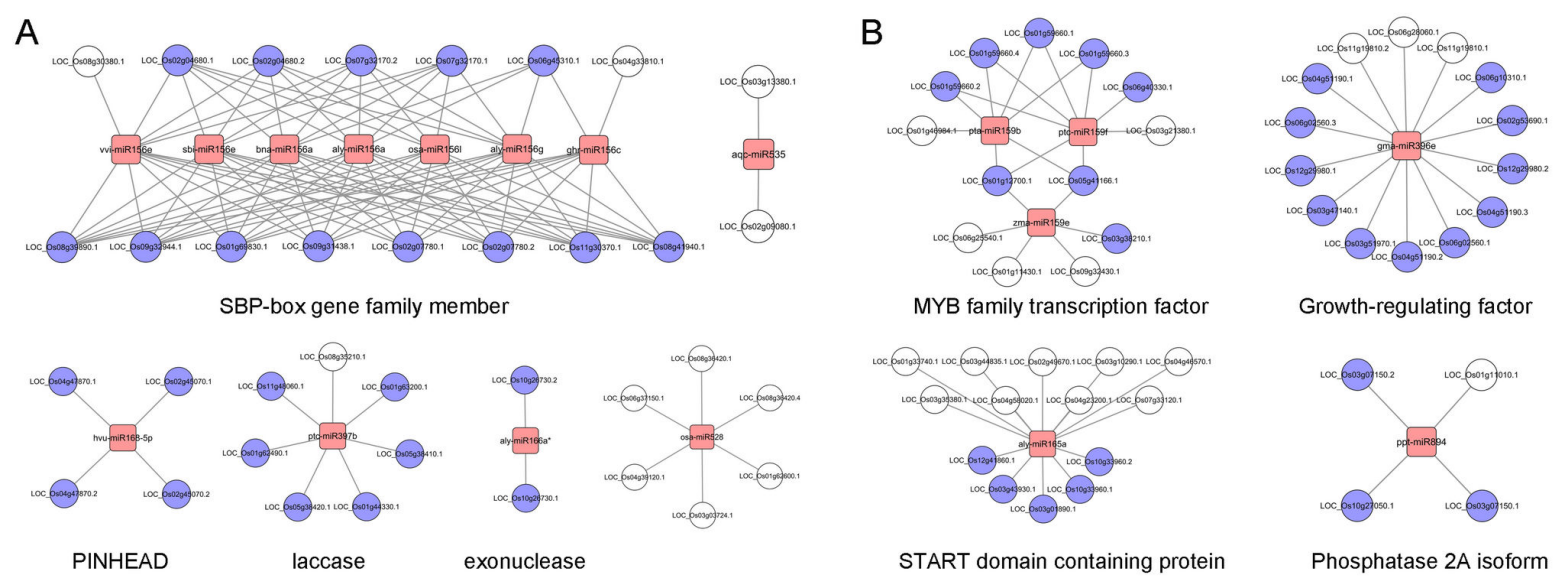
Differential-expression microRNA-mediated regulatory networks. (A) Network mediated by high expression microRNAs in *P* *. pedatisecta* . (B) Network mediated by low expression microRNAs in *P* *. pedatisecta* . The predicted regulatory relationships between miRNAs and targets were denoted by blank lines. The targets of blue nodes were annotated at the bottom and all the networks were constructed using Cytoscape [35].

Based on the obtained miRNA–target pairs, the miRNA-mediated regulatory networks were constructed using Cytoscape [38]. According to their miRNA expression patterns, these networks could be classified into two categories (Figure 4). The results support the well-known miRNA-mediated regulation. For example, the miR156 family was more abundant in *P* *. pedatisecta* , and different members of this family were found to regulate the SPL genes (LOC_Os06g45310, LOC_Os08g39890, LOC_Os11g30370, and so on) from the SBP-box gene family (Figure 4A and Table S1) which consistent with the previous reports [37,39,40]. The genes LOC_Os05g41166 and LOC_Os01g12700 belonging to MYB family transcription factor were identified as targets of the miR159b, miR159e, and miR159f that influence flowering and male fertility [7]. Other miRNA targets are involved in diverse physiological and metabolic processes including growth regulating factor, laccase, START domain containing protein, growth regulating factor protein, ATPase-like domain containing protein, PP2Ac, and PWWP domain containing protein (Table S1).

### Functional analysis of the miRNA targets

**Figure 5:**
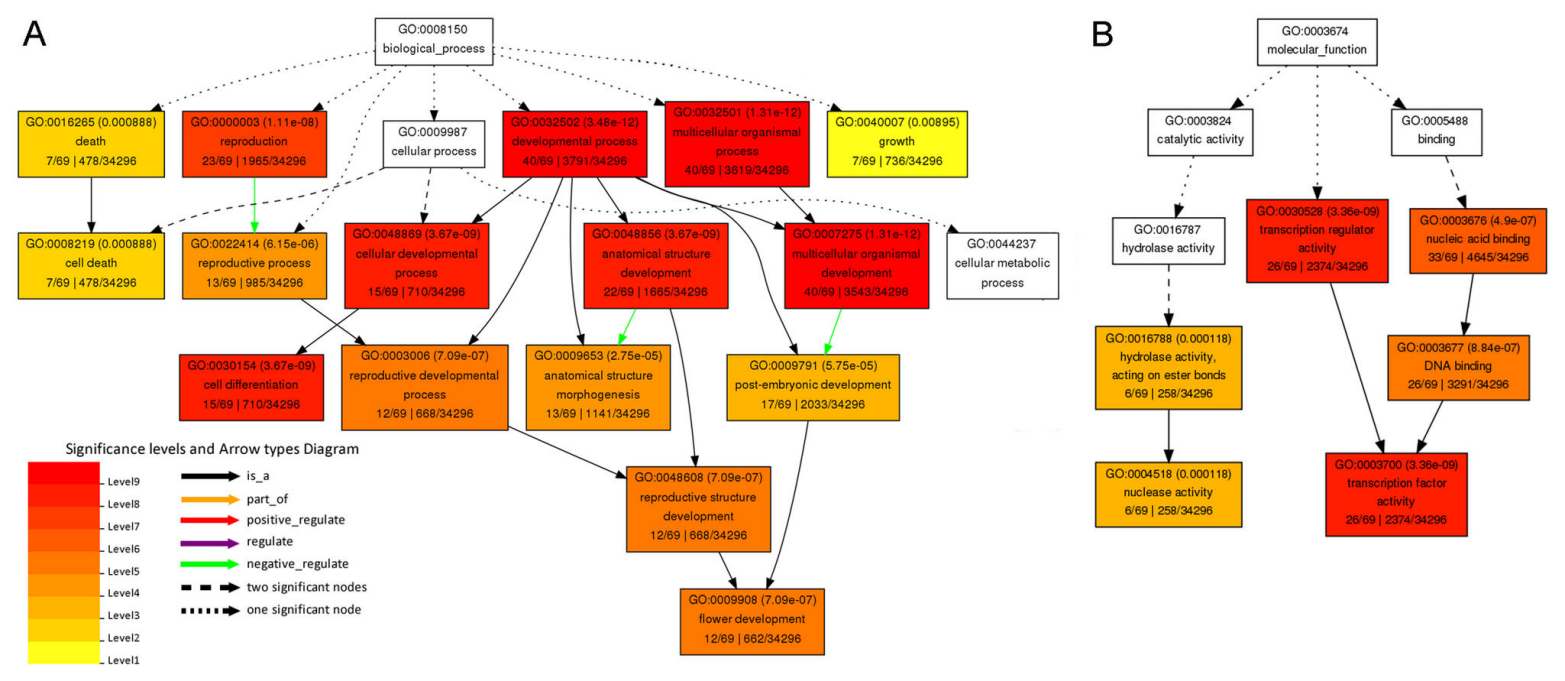
GO (Gene Ontology) term enrichment analysis of the target genes of differential expression miRNAs between *Pinellia* *ternata* and *Pinellia* *pedatisecta* . (A) Analysis of the targets of the differential expression microRNAs within the ‘“Biological Process”’ category. (B) Analysis of the targets of the differential expression microRNAs within the “Molecular Function” category. This analysis was performed by using the online tool agriGO, selecting the “Oryza sativa MSU 7.0” as a background.

To better understand the functional roles of predicted miRNA target genes, we subjected these genes to functional enrichment analysis by using AgriGO, a promising method for uncovering the miRNA-gene regulatory network on the basis of biological process and molecular function [41] (via ‘‘Singular Enrichment Analysis’’; species, *Oryza sativa* MSU 7.0; reference, Rice MSU 7.0 nonTE transcript). The genes targeted by differentially expressed miRNAs were strongly associated with development (57.97%), transcription factor activity (37.68%), reproduction (33.33%) and cell differentiation (21.74%) (Table S2). Many miRNA families were involved in the same biological process. For example, miR156, miR168 and miR396 participated in “multicellular organismal development” within the “Biological Process” category (Figure 5A). while the target genes of miR156, miR159, and miR165 were enriched with the GO term “transcription factor activity” within the “Molecular Function” category (Figure 5B). In addition, We observed that the enriched GO terms ‘‘reproductive structure development,’’ ‘‘anatomical structure development,’’ ‘‘post-embryonic development,’’ and ‘‘multicellular organismal process” were potentially related to ‘‘organ development’’. It indicated that these differentially expressed miRNAs were associated with organ development in *Pinellia* .

### Identification of miRNA targets by degradome sequencing

Accurate validation of miRNA targets is important to thoroughly reveal the biological roles of miRNAs. The degradome sequencing approach was applied to identify the gene targets of *P* *. pedatisecta* miRNAs and elucidate the potential biological functions of its miRNAs [17]. A total of 19 429 349 raw reads, with 93% (18 137 269) of the sequences having the 5′ ends of uncapped, polyadenylated RNAs were generated.

**Figure 6:**
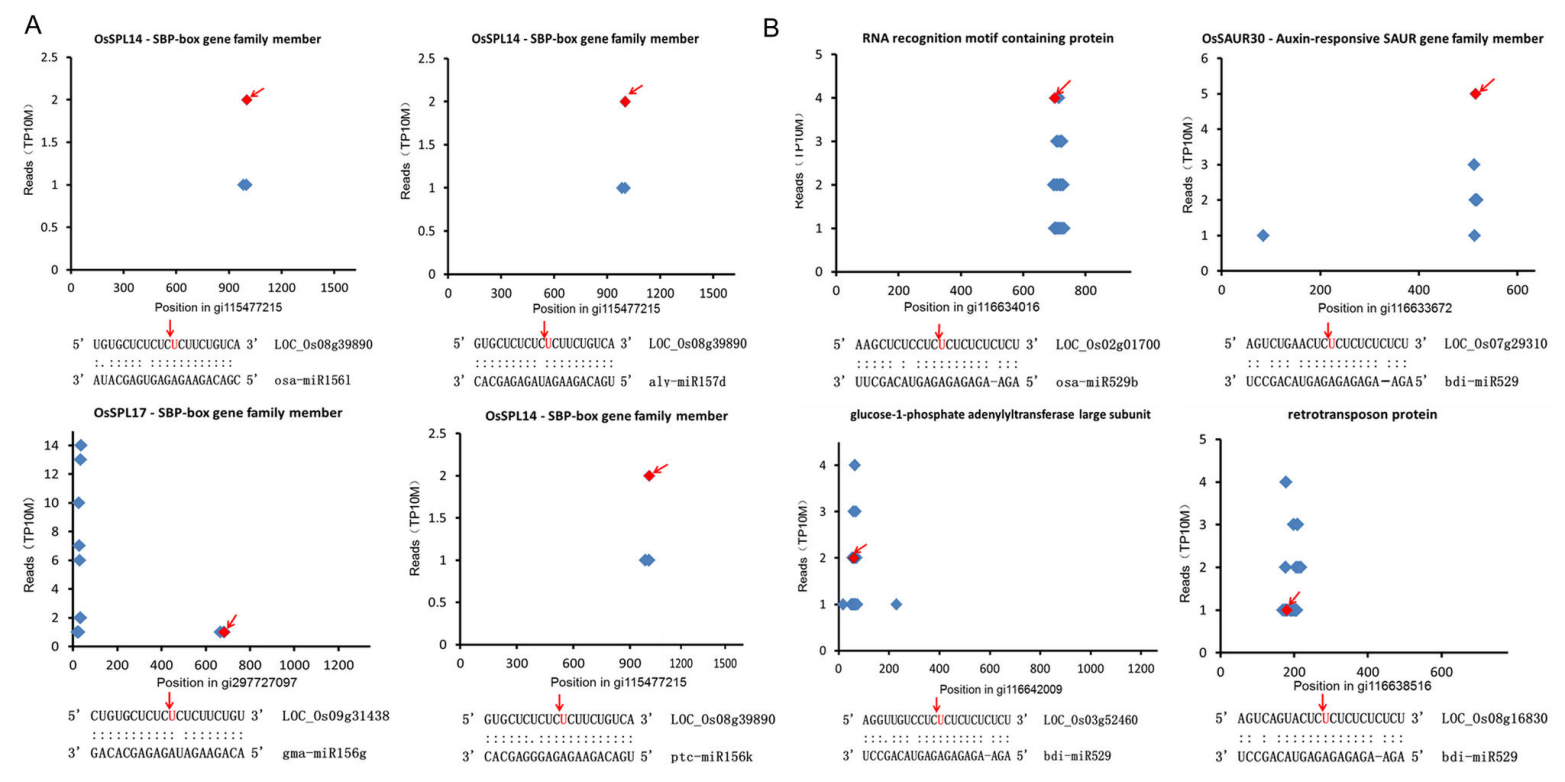
Identified miRNA targets by degradome sequencing are presented in the form of t-plots. The T-plots which were referred to as ‘target plots’ by German et al indicated the distribution of the degradome tags along the sequences of targets (top). The alignment of mRNA: miRNA show the matching degree between miRNA and its target sequence (bottom). The two dots indicate matched RNA base pairs; one dot indicates a GU mismatch. The red spot and arrow represents the sliced target site. The y-axis measures the intensity of the cleavage signal, and the x-axis indicates the position of the cleavage signal on a speciﬁc transcript. (A) Regulation of SBP-box gene family by miR156 and miR157 family identified by degradome sequencing. (B) Different target genes cleaved by the miR529 family.

We used the rice genome for the degradome sequencing study. After removing the respective adaptor sequences and pairing these with *O. sativa* cDNA, 29% (5 328 833) of the distinct degradome reads from 35 430 cDNAs were perfectly matched to the rice genome. Subsequently, the 15 nt upstream and downstream of these mapped cDNAs sequences were extracted to generate the 30 nt target signatures, which were designated as “t-signatures.” These t-signatures were collected to identify and classify the cleaved miRNA targets by the CleaveLand pipeline [42]. The targets of 18 miRNAs from 4 miRNA familieswhich identified by the *Pinellia* miRNA double-color microarray were detected (Table 2). 7 members of miR156 family and miR157d were identified tocleave the same gene: LOC_Os08g39890, which encodes for SPL14 (SBP-box gene family). Furthermore, The result showed that miR156g could target LOC_Os09g31438 which encodes for SPL17. miR529 has four types of potential gene targets, including glucose-1-phosphate adenylyltransferase, SAUR30 (an auxin-responsive member of the SAUR gene family), RNA recognition motif-containing proteins, and retrotransposon proteins (Figure 6). miR529 apparently performs its additional functions in *Pinellia* by targeting multiple genes.

**Table 2:** *P* *. pedatisecta* miRNA targets identified by degradome sequencing.

**miRNA family**	**miRNA Name**	**mRNA annotation**	**RAP_ID**	**Alignment Score**	**Alignment Range**	**Cleavage Site**	**Category**
miR156	osa-miR156l	OsSPL14-SBP-box gene family member	LOC_Os08g39890	2.5	991-1011	1002	I
	bna-miR156a	OsSPL14-SBP-box gene family member	LOC_Os08g39890	1	991-1011	1002	I
	vvi-miR156e	OsSPL14-SBP-box gene family member	LOC_Os08g39890	2	992-1011	1002	I
	ppt-miR156a	OsSPL14-SBP-box gene family member	LOC_Os08g39890	1	992-1011	1002	I
	sbi-miR156e	OsSPL14-SBP-box gene family member	LOC_Os08g39890	1	992-1011	1002	I
	ptc-miR156k	OsSPL14-SBP-box gene family member	LOC_Os08g39890	0.5	992-1011	1002	III
	vvi-miR156h	OsSPL14-SBP-box gene family member	LOC_Os08g39890	0.5	992-1011	1002	I
	gma-miR156g	OsSPL17-SBP-box gene family member	LOC_Os09g31438	2	673-692	683	III
miR157	aly-miR157d	OsSPL14-SBP-box gene family member	LOC_Os08g39890	2	992-1011	1002	I
miR529	bdi-miR529	glucose-1-phosphate adenylyltransferase large subunit	LOC_Os03g52460	3.5	45-66	56	II
	bdi-miR529	OsSAUR30-Auxin-responsive	LOC_Os07g29310	4	500-521	511	II
	bdi-miR529	OsSAUR30-Auxin-responsive	LOC_Os07g29310	4	504-525	515	I
	bdi-miR529	Retrotransposon protein, putative, SINE subclass	LOC_Os08g16830	4	169-190	180	III
	aqc-miR529	glucose-1-phosphate adenylyltransferase large subunit	LOC_Os03g52460	4	45-66	56	II
	far-miR529	glucose-1-phosphate adenylyltransferase large subunit	LOC_Os03g52460	4	45-66	56	II
	osa-miR529b	glucose-1-phosphate adenylyltransferase large subunit	LOC_Os03g52460	4	45-66	56	II
	osa-miR529b	RNA recognition motif containing protein	LOC_Os02g01700	4	690-711	701	I
miR319	zma-miR319a-5p	retrotransposon protein	LOC_Os01g57960	3.5	662-681	672	III

**Figure 7:**
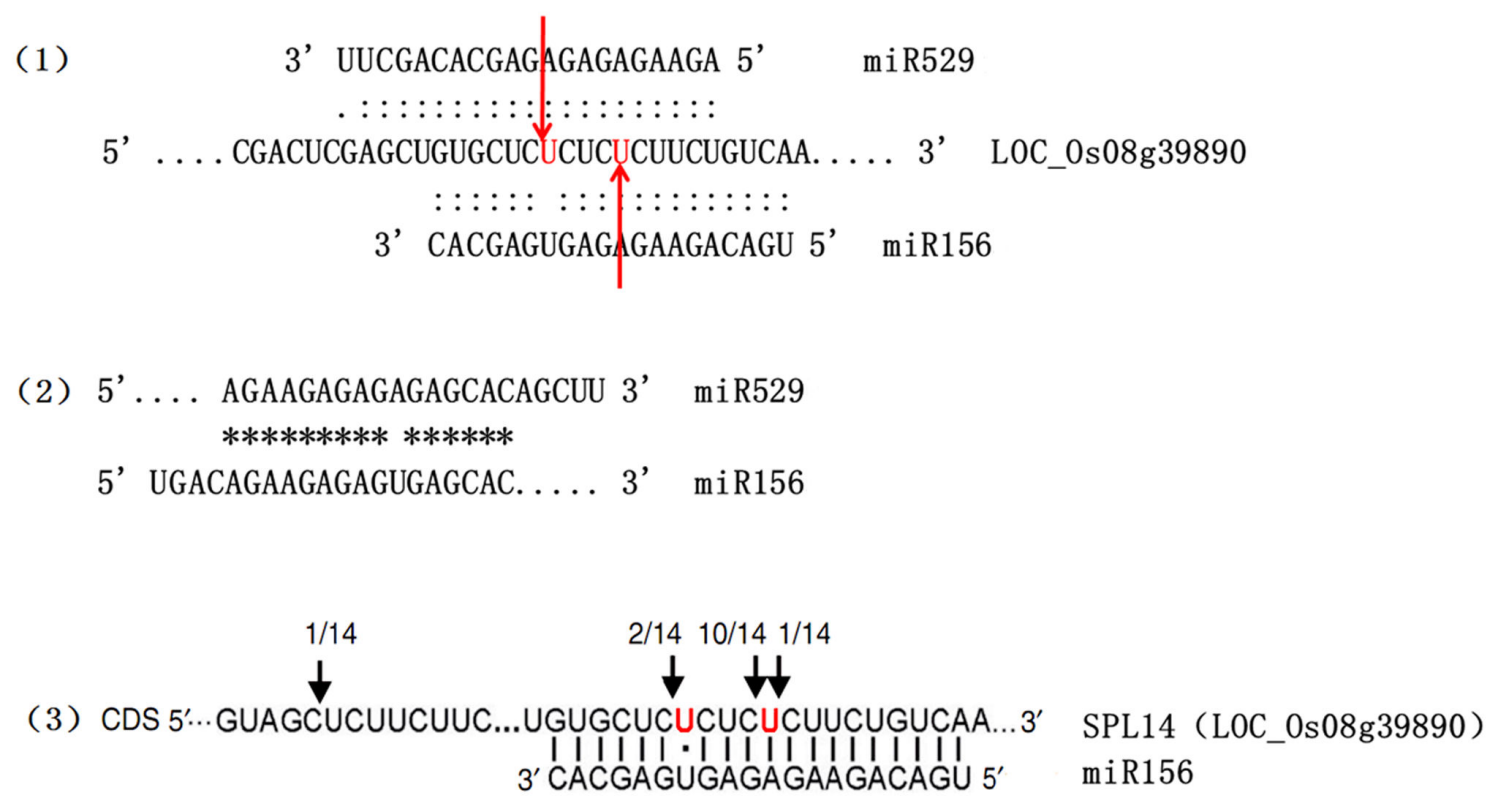
Cleavage analyses with respect to SPL14 targeted by miR529 and miR156. (1) SPL14 mRNA(LOC_Os08g39890) aligned with miR529 and miR156. (2) miR529 and miR156 sequence alignments. *means homology sequence(3). Cleavage sites of SPL14 transcript by miR156 were determined by Jiao et al using 5’ RACE assay. The number of clones which represent the cleavage frequencies is indicated above the sequence.

To determine whether SPL14 could be regulated by miR156, we compared the present results with those reported in the literature. The SPL14 target of miR156 was previously validated by experiments of several independent laboratories [43,44,45,46]. Jiao [45] mapped the miR156-directed cleavage sites of SPL14 using 5′ RACE. The sequencing of 14 randomly chosen clones showed that 13 clones had 5′ ends of the cleaved fragments in the central region of the miR156 target site, thereby confirming that SPL14 can be precisely cleaved by miR156. However, miR529 shares a 14 nt to 16 nt sequence that is significantly homologous with miR156. Given the high sequence similarity, miR529 was predicted to cleave the same genes LOC_Os08g39890 and LOC_Os09g31438 at an overlapping site. By considering that miRNA-guided cleavage occurs precisely between the 10th and 11th nucleotide from the 5′ end of miRNA, we analyzed the results of 5′ RACE by Jiao et al. [45]. We speculated that two clones were generated during 5′ RACE by miR529 but not by miR156 (Figure 7). SPL14 in plants may be targeted by the combined activity of both miR156 and miR529. In addition, miR156 predominantly regulates SBP transcription. However, whether miR156a and miR529b target the same gene SPL14 remains to be verified by more methods.

## Discussion

miRNAs widely exist in animals and plants. Increasing evidence demonstrates that miRNAs have critical roles in regulating development, hormone response, stress response, and other biological processes [47]. Although an increasing amount of plant miRNAs are registered in the miRBase database, miRNA studies on *Pinellia* are very limited. Given the special growth and reproduction mechanisms of *P* *. pedatisecta* , the identification of *P* *. pedatisecta* miRNAs and their targets will greatly advance current knowledge on the physiological functions of miRNAs. In the present study, 101 unique plant miRNAs from 22 miRNA families were identified in *P* *. pedatisecta* and *P* *. ternata* by miRNA microarray analysis.

Based on the plant miRNA conservation across 71 plant species, Zhang et al. [48] classified miRNA into four classes (highly conserved, moderately conserved, lowly conserved, and non-conserved). We identified nine highly conserved miRNA families (miR156/157, miR171, miR165/166, miR159/319, miR396, and miR168), three moderately conserved miRNA families (miR164, miR167, and miR397), and eight lowly conserved miRNA family (miR399, miR528, miR894, miR1432, miR1450, miR2919, miR2936, and miR3946). In addition, highly and moderately conserved families have more members than others, thereby suggesting that these miRNAs have important and conserved functions in the plant kingdom. Furthermore, these miRNAs were primarily based on their high homology with the miRNAs of *O. sativa*. It suggested that most of the known miRNA families are highly conserved during the evolution of various plant species. Based on known miRNA sequence information from well-studied plant species, we can apply miRNA-based experimental strategies in plant whose genome has not been fully sequenced.

Ason et al. [49] reported that miRNA conservation does not always indicate conserved expression. Several miRNAs have differential expression levels with absolute conservation of the miRNA sequence between species. Increasing evidence associates miRNA expression to cell proliferation and developmental regulation. Thus, the differential expression of miRNAs between plants probably reflects the changes in growth and differentiation, as well as changes in the overall shape of developing tissues, which leads to their physiological differences [50,51]. Analyzing the special expression patterns of miRNAs between *P* *. ternata* and *P* *. pedatisecta* would provide useful information regarding their regulatory roles in plant physiological processes. Based on signals generated from the miRNA double-color microarray, we compared the miRNA expression profiles of *P* *. ternata* and *P* *. pedatisecta* and discovered 21 differentially expressed miRNAs.

Among the differentially expressed miRNAs, miR156, miR159, miR168, miR396, and miR397 were predicted to target SBP transcription factors, MYB family members, the PINHEAD, GRF, and laccase. The miRNA target genes have been validated in previous experiments; these genes have crucial functions in the growth and development of different plant species. For example, the MYB transcription factors participate in the response to ABA accumulation. Members of the miR159 family redundantly regulate the expression of *MYB33* and *MYB65*. However, only the double mutant mir159ab has pleiotropic developmental defects, including reduced apical dominance and curled leaves. The miR159-controlled MYB regulation is absolutely critical for proper plant development [52]. miR168 overexpression may also affect the accumulation of miR159 which target gene PINHEAD/ZWILLE has overlapping functions with the ARGONAUTE1 gene [53]. ARGONAUTE1 (AGO1) is an RNA endonuclease of the miRNA pathway; its expression is regulated by miR168. It is well established that miR159 was sensitive to perturbations in AGO1 levels. In 35S×2-*mir168a* transformants, the decrease in miR159 accumulation could contribute to leaf adaxialization [33]. Moreover, miR156 targets the SBP-box gene family to regulate floral and leaf growth. *mir156b*-overexpressing transgenic plants results in a shorter plastochron during vegetative growth compared with the wild type, thereby altering the inflorescence architecture and enhancing branching [40]. miRNA397 is predicted to target laccases, which are copper-containing oxidase enzymes found in various plants; these target sites are conserved in *Arabidopsis* , rice, maize, *Populus* , and other plants [32,48]. It is important in plant response to environmental stress, including oxidative stress, drought stress, cold stress, nutrient-deprivation, and copper homeostasis. In addition, Li et al. [49] reported that laccase has an important function in the early morphological formation of somatic embryogenesis, of which miR397 is a key regulator.

To understand the potential biological function of these miRNAs, we used AgriGO to identify 159 target genes of 18 differentially expressed miRNAs, which were mainly involved in 36 physiological processes. The vast majority of enriched GO terms are related to development, transcription factor activity, multicellular organismal process and reproduction. The targets of certain miRNAs are involved in identical physiological processes. Thus, the various miRNAs target different genes but regulate the same processes. For example, miR156, miR159, miR165, miR168 and miR396 probably co-participate in multicellular organismal development, reproduction, anatomical structure development，cell differentiation and transcription factor activity. These results showed that miRNAs in *Pinellia* are involved in developmental regulation and cause mild alterations in the organ phenotypes of *P* *. ternata* and *P* *. pedatisecta* . However, whether these differentially expressed miRNAs alter phenotypes remains to be investigated via gene overexpression or silencing in transgenic plants.

Quantitative real-time PCR is generally more sensitive than microarray and northern blot analysis. This method allows for the absolute quantification of transcript abundance. Thus, qRT-PCR has been used for the independent validation of differentially expressed miRNAs determined by miRNA microarrays. The qRT-PCR data for most differentially expressed miRNAs (miR159f, miR396e, miR397b, miR528, and miR894) showed reproducible correlation with the microarray data. In particular, miR528 was upregulated by sixfold to sevenfold in *P* *. pedatisecta* as compared with *P* *. ternata* , whereas miR159 was downregulated by threefold. Thus, the results were closely correlated with the microarray data. However, the expected upregulation of *miR156g* was not detected in *P* *. pedatisecta* . Similarly, miR535 was upregulated in *P* *. ternata* , but not in *P* *. pedatisecta* . Microarrays face the significant challenge of accurately distinguishing 5’ and 3’ nucleotides including Affymetrix, Agilent, and Illumina microarray platforms [36]. Our study also meet this phenomenon as show in Table 1(miR156 family). We think that the inconsistent expression levels of miR535 and miR156 between microarrays and qRT-PCR will be caused by different sensitivity levels which microarray hardly accurately distinguish the miRNA sequences at the ends of the bases. However, It is also useful to analysis the result of microarray, Because the same family always target the same gene [28].

The accurate identification of miRNA target genes may reveal the regulatory functions of miRNAs. In this study, we identified 18 potential targets for 4 miRNA families in *P* *. pedatisecta* by degradome sequencing. Among these gene targets, 55.6% belonged to category I, thereby indicating that miRNAs are important for cleaving these target genes. It is worth noting that 7 members of the miR156 family could cleave the same gene coding for SPL14 (SBP-box gene family). Meanwhile, miR156g could target another gene (SPL17) of the SBP-box gene family. These findings suggest that the targets of the same miRNA family usually belong to the same gene family. Moreover, we found that the SBP-box gene was cleaved by a pair of miRNAs (miR156 and miR157), in a manner similar to that of *Arabidopsis* [18], grapevine [54], cucumber [55], and other plant species. These data suggested the presence of a combinatorial gene regulation pathway involving a pair of miRNAs in *Pinellia* .

Unfortunately, the target genes of several *Pinellia* -conserved miRNAs were not identified. Similarly, the targets of the non-conserved miRNAs were also limited. This result may be attributed to the lack of complete mRNA data. Moreover, the rice genome was used instead of the *Pinellia* genome, thereby restricting the comprehensive detection of targets to a certain extent. Conversely, miRNAs have essential roles in temporally and spatially coordinating the developmental processes [56]. The lack of analysis for different developmental stages, tissues, organs, or stress conditions may be another reason. In addition, plant miRNAs can regulate their target mRNAs through translational inhibition and transcript cleavage. For example, miRNA172 can base-pair with the mRNA of the floral homeotic gene *APETALA2*; this miRNA silences *APETALA2* expression primarily through translational inhibition [57]. Degradome sequencing cannot detect these translational repression targets.

Overlapping the results between degradome sequencing and prediction, We found that *miR529* which shares a 14 nt to 16 nt sequence of significant homology with miR156 was predicted to cleave the same genes LOC_Os08g39890 and LOC_Os09g31438 at overlapping site. To further study this phenomenon, we analyzed the *miR156*-verified experimental data of 5′ RACE by Jiao et al. [45] and speculated that miR156 predominantly regulates SPL14. However, SPL14 may be targeted by both miR156 and miR529 in special developmental stages or different tissues. This result is consistent with the *tasselsheath4* (*tsh4*) gene, which is a miRNA-targeted SBP-box transcription factor involved in establishing boundaries within the maize inflorescence. Cleavage analysis of *tsh4* transcripts from wild-type tassels by Chuck et al. [44] showed that the majority of cleavage (68%) occurred between base pairs 10 and 11 of the predicted miRNA-binding site of miR529, whereas 31% occurred near the predicted site for miR156. Thus, miR156 and miR529 function together to repress *tsh4* within inflorescences. However, we did not detect any detectable SPL14 cleavages by miR529 in *Pinellia* leaves by degradome sequencing. Three possible reasons may account for these findings. First, miR529 did not demonstrate a predominant role in SPL14 regulation, and the cleaved fragments were too few to be detected. Second, SPL14 may be stringently regulated by miR529 in a specific spatial or temporal expression pattern. Third, SPL14 in *Pinellia* leaves may be targeted by miR156.

In summary, 101 miRNAs from 22 miRNA families were detected in both *P* *. pedatisecta* and *P* *. ternata* , with 21 differentially expressed miRNAs. Based on the computational prediction and GO analysis, we found that these differentially expressed miRNAs were involved in the development of *Pinellia* organs, thereby providing useful information regarding their regulatory roles in plant physiological processes and further enabling us to thoroughly test their roles in *Pinellia* . In addition, We identified 18 potential targets for 4 miRNA families in *P* *. pedatisecta* by degradome sequencing. Based on the experimental data analysis of 5′ RACE by Jiao et al. [45], we speculate that SPL14 in plants may be targeted by both miR156 and miR529 in a combinatorial manner. This report offers a foundation for the further investigation of the functional regulatory roles of miRNAs and their target genes in *P* *. pedatisecta* .

## Materials and Methods

### Plant materials and total RNA extraction

*P* *. ternata* and *P* *. pedatisecta* plants were maintained in a greenhouse at the Zhejiang Sci-Tech University. Leaves with same developmental stages, plant size, and vigor from both *P* *. ternata* and *P* *. pedatisecta* plants were selected and quickly frozen in liquid nitrogen. Total RNA was extracted with the mirVana™ miRNA Kit (Ambion, Austin, TX, USA), according to the manufacturer’s protocol. After quantification, the integrity of the isolated RNA was validated by separating the major rRNA bands on agarose gels.

### miRNA Microarray Design

Based on the known miRNAs in the miRBase database, we designed a miRNA microarray containing 1957 unique plant miRNAs, representing 2521 well-characterized miRNA from 43 plant species (http://microrna.sanger.ac.uk/). Among the 2521 miRNAs, 190 were from *A. thaliana*, 324 from *O. sativa*, 198 from *P* *. patens* , 196 from soybean, 174 from *M. truncatula*, 202 from *Z. mays*, and the remaining 1237 from 37 other plant species. The unique mature miRNAs were spotted onto a µParaflo™ microfluidic microchip using in situ parallel synthesis and RNA hybridization–optimized probes. Each chip contained two replicates of each probe. For microarray quality control, 5S rRNA was designed as the internal positive control, whereas blank and non-homologous nucleic acids served as negative controls. In addition, eight quality control probes were synthesized to perfectly match or to have a single-nucleotide mismatch with eight external spiked-in synthetic RNAs (20-mer).

### Microarray hybridization and data analysis

The miRNA microarray experiment was performed according to the protocol provided by LC Sciences. Total RNA (1 µg) was used as the starting material for microarray assay. The small RNAs (<300 nt) were size-fractionated using a YM-100 microcon centrifugal filter (Millipore) from the total RNA extracted from leaf tissues. After the small RNAs were 3′-extended with a poly(A) tail using poly(A) polymerase, whereas an oligonucleotide tag was ligated to the poly(A) tail for later fluorescent dye staining. To balance hybridization melting temperatures, we chemically modified the detection probes. Hybridization was performed in a µParaflo® microfluidic chip station (LC Sciences) in 100 µL 6× SSPE buffer (0.90 mol/L NaCl, 60 mmol/L Na_2_HPO_4_, and 6 mmol/L ethylenediaminetetraacetic acid; pH 6.8) containing 25% (v/v) formamide; samples were incubated at 34 °C overnight. After hybridization, we washed each microarray in 0.1× SSPE buffer and used the tag-specific Cy3 and Cy5 dyes to label *P* *. ternata* and *P* *. pedatisecta* .

The hybridization images were digitized using an Axon Gene Pix 4000B Microarray Scanner (Axon/Molecular Device, Sunnyvale, CA, USA) and analyzed using the Array-Pro image analysis software (Media Cybernetics). Raw data were used by first subtracting the background and then normalizing the signals with a locally weighted regression (LOWESS) filter (miRNA microarray data were deposited into the NCBI-GEO with accession no. GSE48322). Two criteria were used to accept a miRNA signal as detectable: (1) a signal intensity higher than three times the background standard deviation, and (2) spot CV < 0.5 (where CV = signal standard deviation/signal intensity). Signals from *P* *. ternata* and *P* *. pedatisecta* plants were compared using the paired, two-tailed Student’s *t*-test; only signals with *p* < 0.05 and differential expression greater than a threefold increase or decrease were considered significant (Cebeci and Budak, 2009).

### Computational prediction of miRNA targets

To predict the genes targeted by differentially expressed miRNAs, three computational target prediction algorithms (psRNAtarget http://plantgrn.noble.org/psRNATarget/, Targetfinder and WMD3 http://wmd3.weigelworld.org/cgi-bin/webapp.cgi?page=TargetSearch;project=stdwmd) were used. The miRNA sequences were reverse-complemented and matched against the Rice MSU (version 7.0) mRNA database. Each mismatch is converted to a weighted score according to the criterion in Jones-Rhoades and Bartel [58]. Mismatched pairs or single nucleotide bulges scores 1, and G:U pairs scores 0.5. The mismatched and G:U pair scores were doubled within the core segment to reﬂect the importance of complementarity to the miRNA 5’ end for target site function [59]. Perfect matches were given a score of “0”, and a cutoff score of “4” was selected to minimize the number of non-authentic targets. The data predicted by these algorithms were combined and the overlaps were calculated(Table S1).

### Expression validation of miRNA using qRT-PCR

Quantitative RT-PCR was used to validate the differences in the expression levels of miRNAs in *P* *. pedatisecta* and *P* *. ternata* . Total RNA was isolated from the leaves of *P* *. pedatisecta* and *P* *. ternata* and treated with RNase-free DNase I to remove DNA contamination from the RNA. These samples were simultaneously collected with those for degradome sequencing. Based on the microarray results, 7 miRNAs with expression levels showing at least a 1.5-fold change were selected, and primers were designed according to Varkonyi-Gasic et al. [60] (Table S3). Total RNA (250 ng) was reverse-transcribed into the miRNA cDNA by RTase M-MLV (Takara Bio, D2639A) using stem-loop primers by following the manufacturer’s protocol. qRT-PCR with SYBR Green (Invitrogen, 11733-038) was performed using the Applied Biosystems 7900 apparatus with the following cycling parameters: 50 °C for 2 min, 95 °C for 2 min, followed by 40 cycles of denaturing at 95 °C for 15 s and annealing at 60 °C for 30 s, with a plate read between each cycle. A disassociation stage (melting curve analysis) was then performed. The threshold cycle (*C*_t_) was defined as the cycle number at which the fluorescence signal exceeded the fixed threshold. For normalized sample *C*_t_ values, the 5.8s rRNA was used (GenBank ID: AF469037.1; forward primer, 5′-GATGAAGAACGTAGCGAAATG-3′; reverse primer, 5′-TCGATGGTTCACGGGATT-3′) was used. The 2^–ΔΔCt^ method was used to analyze the relative changes in gene expression based on the qRT-PCR experiments.

### Degradome library construction and data analysis

To identify the potential target mRNAs, a degradome library using cleaved ends of polyadenylated transcripts was constructed from *P* *. pedatisecta* leaves based on the method described previously by German [17]. Briefly, approximately 1 mg of poly(A)-enriched RNA was directly ligated to a chimeric 5′ RNA adapter (5′-GUUCAGAGUUCUACAGUCCGACGAUCCAGCAG-3′) containing a 3′ Ecop 15I (NEB, R0646S) recognition site using T4 RNA ligase (Promega, M1051). Reverse transcription was carried out to generate first-strand cDNA using an oligo(dT) with a 3′-adapter sequence (5′-CGAGCACAGAATTAATACGACT_(18_)V-3′). Subsequently, a short PCR reaction was used to amplify the cDNA using the primers 5′-CGAGCACAGAATTAATACGACT-3′ and 5′-GTTCAGAGTTCTACAGTCCGACGATCCAG CAG-3′. After purification and digestion with Ecop 15I, a double-stranded 3′-DNA adapter (top, 5′-p-NNTCGTATGCCGTCTTCTGCTTG-3′; bottom, 5′-CAAGCAGAAGACGGCATACGA-3′) with degenerate nucleates in the overhang region was ligated to the Ecop I digestion products using T4 DNA ligase (NEB, M0202S). These products were recovered by PAGE and PCR amplification (18 cycles of 98 °C for 10 s, 60 °C for 30 s, and 72 °C for 15 s.). The final cDNA library was the purified and sequenced using a Solexa/Illumina genome analyzer (LC Sciences, Hangzhou, China). Raw sequencing reads were obtained using the Illumina Pipeline (version 1.5) software to remove adaptor sequences and low-quality reads. In addition, to easily analyze the miRNA targets and RNA degradation patterns, t-plots were built according to the distribution of signatures (and abundances) along these reads [61]. The identification and classification of categories of the sliced miRNA targets were processed according to the CleaveLand pipeline (Axtell Laboratory, Pennsylvania State University, USA).

## Supporting Information

Table S1 **Prediction results of the target of differentially expressed miRNA**
(XLS)

Table S2 **GO (Gene Ontology) term enrichment analysis of the targets of differentially expressed miRNA.**
(XLS)

Table S3 **Primer sequences used for qRT-PCR.** We thank Nannan Yin, Yu Wang and Bing Lin for recruitment and experimental logistics, Chao Fang and PenG Wu for technical assistance.
(XLS)
